# Implementing a quality improvement initiative for private healthcare facilities to achieve accreditation: experience from India

**DOI:** 10.1186/s12913-023-09619-w

**Published:** 2023-07-27

**Authors:** Tapas Sadasivan Nair, Parvez Memon, Sanjay Tripathi, Ashish Srivastava, Meshach Sunny Kujur, Deepti Singh, Parag Bhamare, Vikas Yadav, Vineet Kumar Srivastava, Suranjeen Prasad Pallipamula, Gulnoza Usmanova, Somesh Kumar

**Affiliations:** 1 Jhpiego - an affiliate of Johns Hopkins University, Prius Platinum, A Wing, 5th Floor, D3, P3B, Saket District Centre, Sector 6, Saket, New Delhi, Delhi, 110017 India; 2 Jhpiego - an affiliate of Johns Hopkins University, Lucknow, Uttar Pradesh India; 3 Jhpiego - an affiliate of Johns Hopkins University, Ranchi, Jharkhand India

**Keywords:** Accreditation, Private sector, Quality of care, Maternal health

## Abstract

**Background:**

The Manyata program is a quality improvement initiative for private healthcare facilities in India which provided maternity care services. Under this initiative, technical assistance was provided to selected facilities in the states of Uttar Pradesh, Jharkhand and Maharashtra which were interested in obtaining ‘entry level certification’ under the National Accreditation Board for Hospitals and Healthcare Providers (NABH) for provision of quality services. This paper describes the change in quality at those Manyata-supported facilities when assessed by the NABH standards of care.

**Methods:**

Twenty-eight private-sector facilities underwent NABH assessments in the three states from August 2017 to February 2019. Baseline assessment (by program staff) and NABH assessment (by NABH assessors) findings were compared to assess the change in quality of care as per NABH standards of care. The reported performance gaps from NABH assessments were then also classified by thematic areas and suggested corrective actions based on program implementation experience.

**Results:**

The overall adherence to NABH standards of care improved from 9% in the baseline assessment to 80% in the NABH assessment. A total of 831 performance gaps were identified by the NABH assessments, of which documentation issues accounted for a majority (70%), followed by training (19%). Most performance gaps could be corrected either by revising existing documentation or creating new documentation (62%), or by orienting facility staff on various protocols (35%).

**Conclusion:**

While the adherence of facilities to the NABH standards of care improved considerably, certain performance gaps remained, which were primarily related to documentation of facility policies and protocols and training of staff, and required corrective actions for the facilities to achieve NABH entry level certification.

**Supplementary Information:**

The online version contains supplementary material available at 10.1186/s12913-023-09619-w.

## Background

Accreditation is an important strategy to improve and assure the quality of health care [1, 2]. Accreditation has been defined by the International Society for Quality in Healthcare (ISQua) as, “A public recognition by a healthcare accreditation body of the achievement of accreditation standards by a healthcare organization, demonstrated through an independent external peer assessment of that organization’s level of performance in relation to the standards” [1]. Over the last 30 years, ISQua has been working to promote quality improvement in healthcare by developing the quality care standards and recognizing various accreditation standards and programs [3].

As a strategy to improve the quality of services and patient outcomes in public healthcare facilities [4], the Government of India has introduced initiatives targeting various aspects of quality in service delivery and facility operations of public sector health facilities, including the *Kayakalp* (transformation) initiative in 2015 [5], the *Dakshata* (adroitness) program in 2015 [6], the National Quality Assurance Standards in 2016 [7], the Labour Room Quality Improvement initiative (*LaQshya*) in 2017 [8], development of standard treatment guidelines [9], and the *Ayushman Bharat* program in 2018 [10, 11]. However, in India, the private sector has not received similar attention despite contributing to 80% of general outpatient care, 60% of inpatient care [12], and up to 30% of institutional deliveries in rural areas and 52.5% of institutional deliveries in urban areas [13]. Existing evidence indicates that the quality of maternity care in private healthcare facilities in India is suboptimal [4, 14–22]. In the absence of any comparable quality improvement initiative mandated for the private sector, quality assurance in private-sector health facilities in India remains largely voluntary. Considering the key role played by the private sector in the country, incentivizing facilities to get accreditation could facilitate the improvement of quality of care and the achievement of universal health coverage [1, 23, 24].

Presently in India, the principal hospital accrediting bodies are the National Accreditation Board for Hospitals and Healthcare Organizations (NABH), the Joint Commission International, Bureau Veritas International, and the International Organization of Standards. Among these, NABH is widely known and has been specifically developed for the Indian setting [25]. NABH was established in 2006 under the Quality Council of India with the vision of becoming the apex national healthcare accreditation and quality improvement body in the country. The standards of accreditation under NABH have been recognized by ISQua [26].

NABH has defined accreditation standards for different types of health facilities. Facilities with up to 50 beds are considered small health care organizations (SHCOs) [27], while larger facilities are categorized as hospitals [28]. NABH also has defined three different levels of accreditation — ‘entry level certification’, ‘progressive level certification’, and ‘full accreditation’ — and hospitals can apply for the level of their choice. In 2016, the Insurance Regulatory and Development Authority of India notified providers offering cashless mediclaim services (health insurance coverage for hospitalization) that they must meet entry-level certification standards laid down by NABH [29]. In 2018, the *Ayushman Bharat Pradhan Mantri Jan Arogya Yojana* (AB-PMJAY) – a national health assurance scheme under the *Ayushman Bharat* program – was launched, in which empaneled hospitals are eligible to receive reimbursements at higher rates if they are accredited under NABH [30]. Thus, the potential increase in clientele and revenues that may result from getting accreditation has become a major incentive for private healthcare facilities to achieve the quality standards [25, 31].

### Program description

**Fig. 1:**
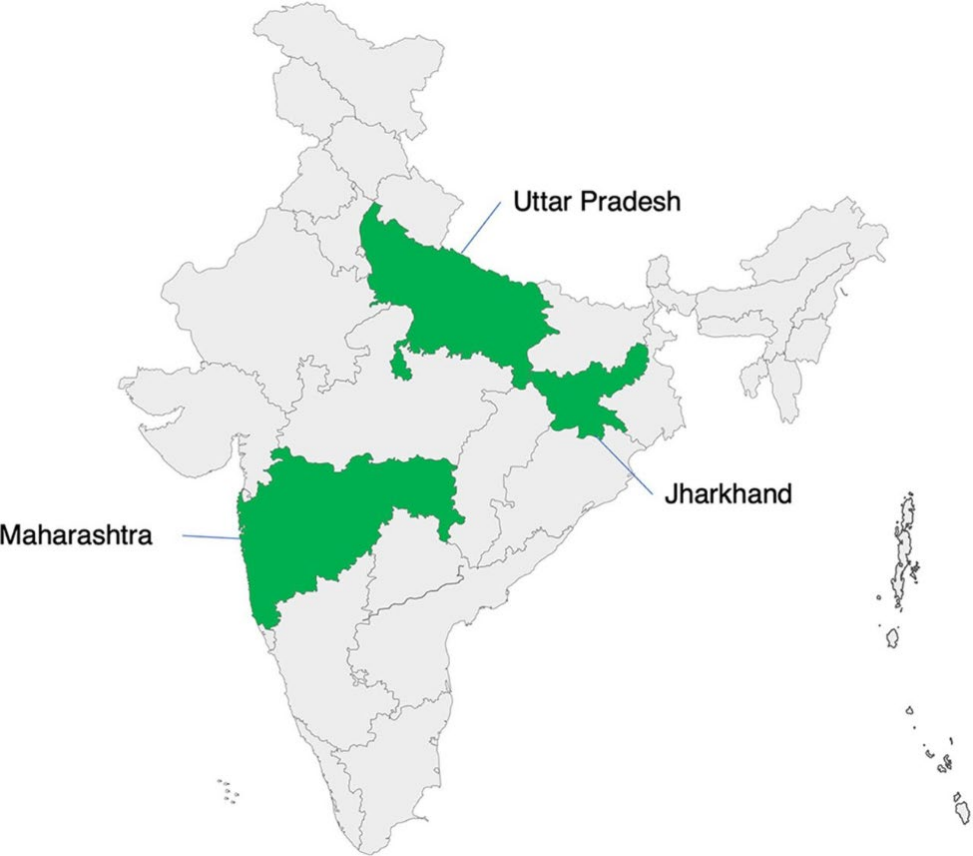
Manyata program intervention states

The Manyata (Hindi for “accreditation” or “recognition”) program is a quality improvement and certification initiative offered for private healthcare facilities by the Federation of Obstetric and Gynaecological Societies of India (FOGSI), which is being implemented by Jhpiego (an international non-profit health organization affiliated with the Johns Hopkins University) with funding support of MSD for Mothers [15, 32]. Based on the World Health Organization’s Safe Childbirth Checklist which was adapted under the guidance of FOGSI and with technical assistance from Jhpiego, the program is centered on 16 quality standards for maternity care which were considered to be achievable at small private healthcare facilities, including antenatal care, timely identification and management of complications, adherence to infection prevention protocols, cesarean deliveries and respectful maternity care. After an initial phase of piloting, the program was officially launched in 2016 in three states - Uttar Pradesh, Jharkhand, and Maharashtra (Fig. 1).

### Program activities

The Manyata program relied on the FOGSI’s leadership and network (local societies) to spread awareness and encourage involvement among its membership through district-level sensitization meetings. Facilities, which voluntarily opted to participate in the program, underwent a baseline assessment by Jhpiego program officers (who were either nursing professionals or doctors) using the Manyata checklist, based on the 16 standards for maternity care. These facilities then received three days training on skills and competencies related to key evidence-based practices for care during childbirth and structured mentoring support visits for a period of four-six months to ensure the translation of skills to practice. Following this, they were deemed “Manyata certified” if they achieved a set number of standards in the final assessment, which was conducted by a FOGSI assessor.

As part of Manyata program implementation, it was decided to provide strategic technical support to those facilities which were interested in applying for NABH entry level certification, considering the interest among and motivation for private healthcare facilities to get accreditation with NABH [25, 29–31]. The facilities which were interested in NABH accreditation underwent a baseline assessment by Jhpiego program officers using the NABH checklist. The program officers identified performance gaps in practices, procedures and other reasons for a facility’s non-adherence to quality standards; and worked with facility staff to develop action plans and implement strategies to address those gaps. These facilities then received dedicated preparatory support in the form of a one-day managers’ orientation and two days staff training on hospital standards, in addition to the classical quality improvement package of trainings and mentoring support visits under the Manyata program. Further, during the mentoring support visits, program officers supported the facilities over a period of two-six months (depending on the facilities’ baseline level of preparedness) for the development of standard operating procedures; establishment of in-facility training and human resource, medication, and information management systems; and tracking progress over time. Program officers also supported the facility in the application process and facilitated the NABH assessments, which were carried out independently by NABH assessors (who are either medical, nursing, or healthcare management professionals, and have been trained through a standardized five-day training in accordance with the guidelines mentioned in the assessors’ handbook [33].

**Fig. 2:**
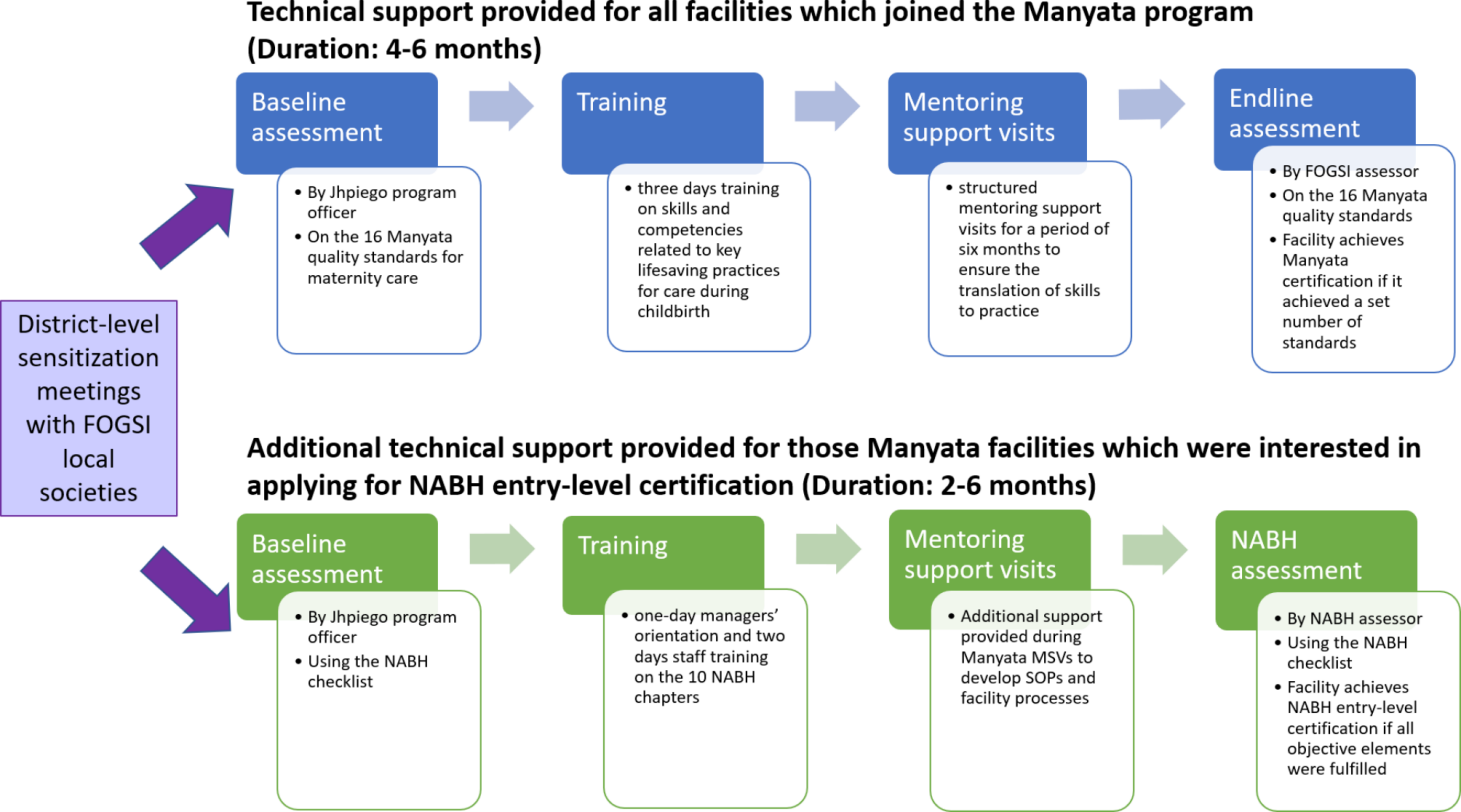
Activities and timelines for Manyata program implementation and for NABH entry-level certification

The activities and timelines for the implementation of Manyata program as well as the additional technical support provided to facilities for NABH entry-level certification are shown in Fig. 2.

This paper aims to describe the change in quality of care in Manyata supported facilities when assessed by the NABH standards of care. It intends to yield insights about the performance gaps in private healthcare facilities and the corrective actions required for facilities to receive NABH entry level certification.

## Methods

Out of the 383 private healthcare facilities that had participated in the Manyata program, 102 facilities across the three states applied for NABH entry level certification from October 2016 till November 2018. Analysis in this paper includes the baseline assessments and NABH assessments data for the 28 private healthcare facilities that underwent NABH assessments between August 2017 and February 2019.

### Assessment Tool

**Fig. 3:**
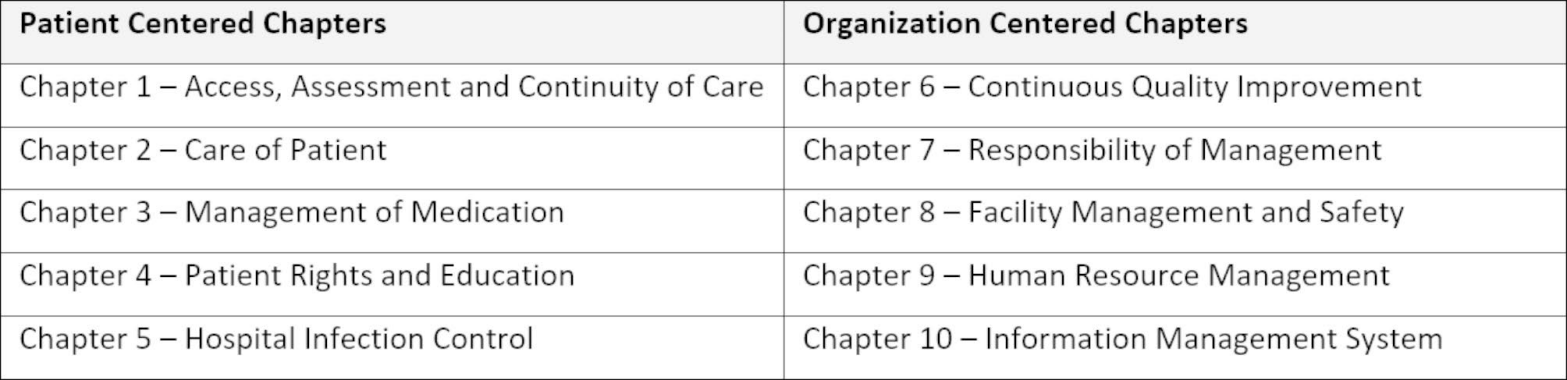
NABH chapters in the entry level certification checklist

The tool utilized for assessment is the standardized NABH checklist for assessing adherence to prescribed standards. NABH standards are organized into 10 chapters; the first five are patient centered and the last five are organization centered (Fig. 3).

Each chapter has standards that are broad statements of care. Each standard has objective elements against which compliance is validated in a systematic manner. The entry-level certification checklist differs slightly for SHCOs and hospitals, with the hospital checklist being slightly more detailed. While the NABH checklist for SHCOs has 10 chapters, 41 standards, and 149 objective elements, the checklist for hospitals has 10 chapters, 45 standards, and 167 objective elements [27, 28] (Supplementary Material S1 and S2).

### Assessment process

The assessment process during the baseline assessments and the NABH assessments was the same; both sets of assessors were trained on the methodology using the same approach. The assessment process involved: (a) the review of hospital policies and protocols on the basis of the facilities’ scope of services (known as *documentation*), (b) physical verification of resources and equipment and anonymized key informant interviews with providers (known as *implementation)*, and (c) review of the completeness of case records (known as *evidence*). The assessors evaluated the relevant documentation, implementation, and evidence for each objective element in the NABH checklist in same manner. To determine whether the facility adhered to a prescribed standard, the assessor first reviewed the facility’s policies and standard operating procedures (SOPs) for their relevance, comprehensiveness, consistency with standard treatment guidelines, and alignment with the scope of services. If the policies and SOPs were found to be incomplete or missing for any objective element, the assessor would mark the objective element as “non-compliance”. Hence, the score would be zero and the assessor would not further delve into the corresponding implementation and evidence for that objective element. If the assessor felt that the policies and SOPs were in line with the scope of services, the implementation of the defined policies and SOPs would be assessed by interviewing the relevant facility staff through unstructured interviews and verifying the presence of needed equipment and other resources. The assessor would also review a sample of case records through convenience sampling method to check for evidence of implementation. If any gaps were observed in either the implementation or evidence, the assessor would mark the objective element as “non-compliance” and the score would be five. If the documentation, implementation and evidence was available for the objective element, then the score would be ten. As part of NABH routine practice, the NABH assessor shared the completed assessment form along with a list of non-compliances with the facility team for corrective action. The facility was then required to submit a report to the NABH within a defined timeframe documenting the changes they have made to address the non-compliances in order to achieve NABH entry level certification. The facility was awarded NABH entry-level certification when all the objective elements were fulfilled.

### Data analysis

The private healthcare facilities were profiled in terms of geographical location, number of beds, monthly delivery load and type of facility. A de-identified dataset containing the facilities’ baseline and NABH assessment findings was collated, compiled and analysed in Microsoft Excel 2016 by NABH chapter and objective element. Facility scores were calculated for baseline and NABH assessments based on the NABH checklist, in terms of the percentage of objective elements achieved. The most commonly reported non-compliances during NABH assessments were listed. The distribution of non-compliances was then presented in terms of broad thematic areas and required corrective actions.

After a review of the consolidated list of non-compliances from the NABH assessment results of the 28 healthcare facilities, the authors grouped them into the following five broad thematic areas based on their program implementation experience: *Documentation* non-compliances related to lack of documentation or improper documentation, *Licenses and service-level agreements* non-compliances related to memorandums of understanding and licenses for facilities and services, *Manpower* non-compliances related to staffing and human resource management, *Supplies and Maintenance* non-compliances related to the presence of essential resources and the management of the facility environment and equipment, and *Training* non-compliances that result from lack of proper orientation of the hospital staff on various protocols and procedures.

Finally, the authors identified four basic types of actions needed to correct observed non-compliances based on their program experience of working with private healthcare facilities in India. These included revising existing documentation or creating new documentation, orienting staff on procedures and protocols, procuring equipment, and hiring additional staff. They then grouped the non-compliances into the appropriate corrective actions.

### Ethical considerations

All data used in this study were collected as a part of routine monitoring of the Manyata program. All facilities who enrolled in Manyata agreed to take part in monitoring and evaluation activities related to the program. Data were shared back with individual facilities for the purposes of improvement. The Johns Hopkins School of Public Health Institutional Review Board (IRB) deemed the Manyata program activities and routine program data collection to be non-human subjects research, thus not requiring IRB oversight (IRB No: 00009525). The manuscript uses the SQUIRE 2.0 standards for reporting [34].

### Patient and public involvement

Since this was an analysis of health facility assessments data, patients and the public were not involved in any way in this research.

## Results

Of the 28 facilities, 89% were classified as SHCOs while the rest were classified as hospitals, based on their number of beds (Table 1). 82% of the facilities had a monthly delivery load of 50 deliveries or less. 61% of the facilities were categorized as multi-specialty facilities while the remaining were exclusive maternity facilities (39%).

**Table 1:** Characteristics of 28 Manyata-supported facilities that underwent NABH assessments

Characteristic	Number	Percent (%)
**State**		
Jharkhand	5	17.9
Maharashtra	8	28.6
Uttar Pradesh	15	53.6
**Number of beds**		
≤ 50	25	89.3
> 50	3	10.7
**Monthly delivery load**		
< 20	9	32.1
20–50	14	50
> 50	5	17.9
**Type of facility**		
Exclusive maternity facility	11	39.3
Multi-specialty facility	17	60.7

**Fig. 4:**
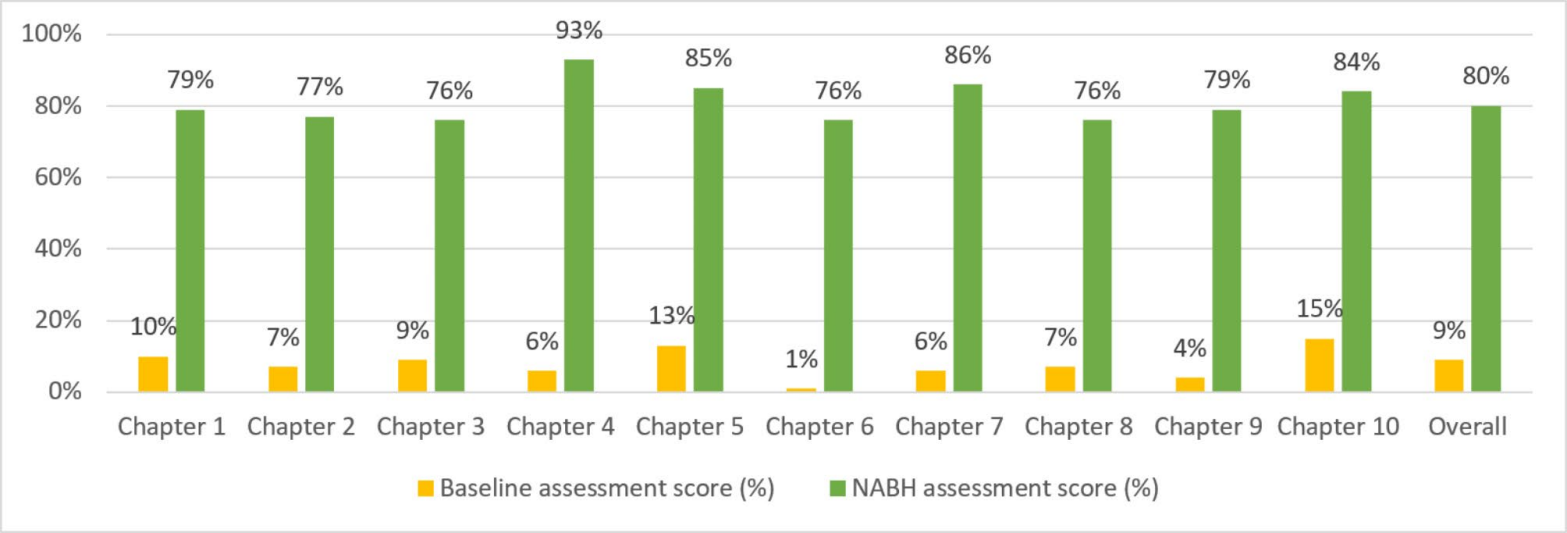
Average facility score from baseline and NABH assessments (n = 28)

The average facility score during baseline assessment (in terms of the percentage of objective elements which were achieved) was 9%, which ranged from 15% for Chap. 10 – Information Management System to 1% for Chap. 6 – Continuous Quality Improvement (Fig. 4). At the time of the NABH assessment, the average facility score improved to 80%. Facilities scored the highest for Chap. 4 – Patient Rights and Education (93%), while they scored the least for Chap. 3 – Management of Medication, Chap. 6, and Chap. 8 – Facility Management and Safety (76% each) (Fig. 4).

The objective elements which showed the maximum improvement from baseline to NABH assessment included the identification of authorized personnel to make entries in medical records, availability of resources for quality improvement programs and availability of documented facility organograms, grievance redressal actions, ensuring the confidentiality of patient information, and the care of patients in consonance with standard treatment guidelines and by qualified personnel who are entitled to perform those procedures. In contrast, the objective elements which showed the minimum improvement included the documentation of medication orders, obtaining informed consent for anaesthesia and surgery, documentation of discharge summaries, availability of imaging results within a defined timeframe, accessibility of hand hygiene facilities for providers in all patient care areas, and the documentation of the scope and content of assessments for in-patients and emergency patients by the health facility (Table 2). Twelve objective elements were achieved by all the facilities, while ten objective elements were not achieved by half or more of the facilities (Supplementary Material S3 Tables 1 and 2).

**Table 2:** Objective elements which showed the maximum and minimum improvement from baseline to NABH assessment

Sl.No.	Name of Objective Element	Number (%) of facilities which achieved the objective element in the baseline assessment	Number (%) of facilities which achieved the objective element in the NABH assessment	% Improvement (from baseline to NABH assessment)
***Objective Elements which showed maximum improvement***				
IMS.1.b	Organization identifies those authorized to make entries in medical record.	0 (0%)	28 (100%)	100%
COP.4.a	Care of patient is in consonance with the documented procedures.	1 (4%)	28 (100%)	96%
COP.8.d	Qualified persons are permitted to perform the procedures that they are entitled to perform.	1 (4%)	28 (100%)	96%
MOM.1.e	Documented procedures address procurement and usage of implantable prosthesis.	1 (4%)	28 (100%)	96%
PRE.1.c	Patient rights include treating patient information as confidential.	1 (4%)	28 (100%)	96%
ROM.2.b	The leaders/management guide the organization to function in an ethical manner.	1 (4%)	28 (100%)	96%
IMS.3.b	Privileged health information is used for the purposes identified or as required by law and not disclosed without the patient’s authorization.	1 (4%)	28 (100%)	96%
CQI.1.c	Hospital Management makes available adequate resources required for quality improvement program.	0 (0%)	27 (96%)	96%
ROM.1.a	The organization has a documented organogram.	0 (0%)	27 (96%)	96%
HRM.2.c	Actions are taken to redress the grievance.	0 (0%)	27 (96%)	96%
***Objective Elements which showed minimum improvement***				
MOM.2.c	Medication orders are clear, legible, dated and signed.	10 (36%)	10 (36%)	0%
COP.7.e	Informed consent for administration of anaesthesia is obtained by the anaesthetist.	3 (11%)	11 (39%)	28%
AAC.6.c	Imaging results are available within a defined time frame and critical results are intimated immediately to the concerned personnel.	8 (29%)	17 (61%)	32%
AAC.7.e	Discharge summary incorporates instructions about when and how to obtain urgent care.	2 (7%)	11 (39%)	32%
IMS.1.d	The author of the entry can be identified.	1 (4%)	11 (39%)	35%
IMS.1.e	The contents of medical record are identified and documented.	11 (39%)	21 (75%)	36%
IMS.1.c	Every medical record entry is dated and timed.	3 (11%)	14 (50%)	39%
COP.8.b	An informed consent is obtained by a surgeon prior to the procedure.	2 (7%)	13 (46%)	39%
HIC.2.a	Hand hygiene facilities in all patient care areas are accessible to healthcare providers.	9 (32%)	21 (75%)	43%
AAC.3.a	The organization defines the content of the assessments for in-patients and emergency patients.	0 (0%)	12 (43%)	43%

NABH assessments reported a total of 831 non-compliances across all 28 facilities (Table 3). Patient-centered chapters accounted for 66% of all non-compliances. The share of the various NABH chapters in the non-compliances did not vary much by state, facility type, by number of beds, or by monthly delivery load (Supplementary Material S3 Table 3). The mean number of non-compliances was 30 for SHCOs (out of 149 objective elements) and 28 for hospitals (out of 167 objective elements). The average number of non-compliances was 32 for exclusive maternity care facilities and 28 for multi-specialty care facilities. However, analysis of the mean non-compliances by state revealed that the average count of non-compliances per facility was 37 in Uttar Pradesh, as compared to 22 each in Jharkhand and Maharashtra.

**Table 3:** Distribution of non-compliances identified during NABH assessments of 28 facilities by NABH chapters

NABH Chapters	Number of Non-Compliances	Percentage (%)
Chapter 2 – Care of Patients	196	23.5
Chapter 1 – Access, Assessment, and Continuity of Care	156	18.8
Chapter 3 – Management of Medication	119	14.3
Chapter 8 – Facility Management and Safety	93	11.2
Chapter 10 – Information Management System	73	8.8
Chapter 9 – Human Resource Management	59	7.1
Chapter 5 – Hospital Infection Control	56	6.7
Chapter 6 – Continuous Quality Improvement	33	4.0
Chapter 7 – Responsibility of Management	28	3.4
Chapter 4 – Patient Rights and Education	18	2.2
**TOTAL**	**831**	

Table 4 shows how non-compliances were distributed across thematic areas. Lapses in documentation accounted for a considerable majority of all non-compliances (70%), followed by training (19%). Documentation was the most prevalent thematic area for non-compliances in all the 10 NABH chapters, while training was the second most common thematic area in eight chapters. Documentation also accounts for nine of the 10 most common non-compliances (Supplementary Material S3 Table 3).

**Table 4:** Distribution of non-compliances by thematic area, according to NABH chapters

NABH Chapters	Thematic Area [n]				
	Documentation	Licenses and Service-Level Agreements	Manpower	Supplies and Maintenance	Training
Chapter 1 – Access, assessment, and continuity of care	115	5	3	2	31
Chapter 2 – Care of patients	145	5	6	7	33
Chapter 3 – Management of medication	83	0	2	6	28
Chapter 4 – Patient rights and education	15	0	2	0	1
Chapter 5 – Hospital infection control	24	5	2	9	16
Chapter 6 – Continuous quality improvement	26	0	3	0	4
Chapter 7 – Responsibility of management	15	8	5	0	0
Chapter 8 – Facility management and safety	46	5	5	11	26
Chapter 9 – Human resource management	44	0	1	1	13
Chapter 10 – Information management system	66	0	0	0	7
**Total number (%)**	**579 (69.7%)**	**28** **(3.4%)**	**29** **(3.5%)**	**36** **(4.3%)**	**159 (19.1%)**

Categorizing the observed non-compliances by the corrective action required shows that most gaps could be resolved either by revising existing documentation or creating new documentation (62%), or by orienting facility staff on various procedures and protocols (35%) (Table 5). It was noted that nearly 26% of the non-compliances classified in the documentation thematic area could be rectified by orienting staff on facility procedures and protocols, while 37% of the non-compliances classified in the training thematic area could be corrected by revising existing documentation or creating new documentation.

**Table 5:** Distribution of non-compliances by type of corrective action required, according to thematic areas

Thematic Area	Number (%) of Non-Compliances by Required Corrective Action			
	Hiring	Revise existing OR create new documentation	Orientation on facility procedures and protocols	Procurement
Documentation	0	431 (74.4%)	148 (25.6%)	0
Licenses and Service-Level Agreements	0	18 (64.3%)	7 (25%)	3 (10.7%)
Manpower	7 (24.1%)	3 (10.3%)	19 (65.5%)	0
Supplies and Maintenance	0	6 (16.7%)	15 (41.7%)	15 (41.7%)
Training	0	59 (37.1%)	100 (62.9%)	0
Total	**7 (0.8%)**	**517 (62.2%)**	**289 (34.8%)**	**18 (2.2%)**

## Discussion

Independent assessments of 28 Manyata-supported private healthcare facilities (25 SHCOs and 3 hospitals) reported an average of 30 non-compliances for SHCOs (out of 149 objective elements) and 28 non-compliances for hospitals (out of 167 objective elements). The average count of non-compliances was 37 for facilities in Uttar Pradesh, as compared to 22 each for facilities in Jharkhand and in Maharashtra. Most of these non-compliances were related to documentation (70%) and training (19%) and could be corrected by revising existing documentation or creating new documentation (62%), or orienting facility staff on various procedures and protocols (35%).

It is worth noting that none of the 28 Manyata-supported facilities were able to achieve entry-level certification during the NABH assessment. They were later able to achieve certification after closure of non-compliances by submitting a report to the NABH documenting the changes they made to address the non-compliances after the NABH assessment. This is probably because the Manyata program focused on clinical standards related to maternity and newborn care, while the NABH certification process focuses on standards for hospital processes and practices for general clinical services, thus highlighting the gap between clinical and hospital standards of care. However, there was a tremendous improvement in the average facility scores in terms of percentage of objective elements achieved, from 9% in the baseline assessment to 80% in the NABH assessment. This reflects the effect of the technical support provided to facilities for NABH entry-level certification as well as the motivation of these facilities to improve their quality of care as per the NABH standards and enhance their facility processes accordingly.

Historically, healthcare facilities that apply for accreditation are tertiary care hospitals seeking better functional and operational efficiency for being part of an accreditation system [35–37]. Documentation of hospital policies, protocols, patient records, and continuous staff trainings — while preventing the overburdening of staff at the same time — were found to be key prerequisites to ensure smooth implementation of the accreditation process [38–43]. At the time of enrollment to Manyata program, we found that private healthcare facilities did not have defined policies and procedures to guide their functioning. However, with the program’s support, facilities developed a set of policies and procedures incorporating evidence-based recommendations for system improvement and client care practices and then oriented facility staff on the newly drafted policies and procedures. The most common non-compliances observed by NABH assessors were related to documentation of medical records, informed consent for surgeries and anaesthesia, medication orders and discharge summaries. This suggests that healthcare facilities require more time and effort to ensure that routine documentation practices are absorbed, implemented, and institutionalized as per the NABH standards.

According to the literature, accreditation is a complex and resource-intensive process that may require healthcare facilities to improve their existing infrastructure and recruit more staff [44–46]. In contrast, this analysis found that most performance gaps identified by NABH assessments were related to ensuring proper documentation at the facility and staff training, while hiring of manpower and procurement of equipment accounted for a minuscule proportion (3% combined) of the observed non-compliances (Table 5). This could be due to the design of the NABH assessment methodology itself, which focuses first on documentation, followed by implementation, and finally on evidence, and thus has resulted in a predominance of documentation-related performance gaps. In addition, while NABH assesses whether processes and practices are implemented, it does not assess the competency of the providers for these practices and processes (“how” they have been implemented).

Although the five NABH patient-centered chapters accounted for two-thirds of reported non-compliances, very few non-compliances were related to patient experience, which is a key determinant of the quality of care [47]. This is probably because the NABH assessment checklist collects client feedback only for certain items, such as informed consent before procedures and billing policy.

Another cause for concern is that while NABH assessors check for completeness of medication orders, there is no provision to check for their correctness or to look for a system of reporting medication errors. In the United States, medication errors are a major threat to the quality of care, leading to as many as 98,000 deaths and costing roughly US$2 billion annually [48]. Hence, accreditation systems need to assess the mechanisms in place at the facility for reporting these adverse events [49].

Globally, it is estimated that one in five births in low- and middle-income countries occurs in the private sector [50]. In India, the private sector accounts for nearly one in every three facility births and thus has an important contribution to the delivery of primary healthcare services [12, 13, 51]. However, existing evidence indicates that the quality of maternity care services provided by the private sector is suboptimal [14–22]. The main contributors to poor quality were lack of qualified staff, unavailability of essential resources, lack of regulatory guidelines, lack of adherence to standard treatment guidelines, and absence of quality improvement initiatives. In this context, the Manyata program plays a valuable role in improving quality of care by building the capacity of the service providers for delivering maternity care services, improving the availability of essential resources, enhancing the adherence to standard treatment guidelines, and inculcating a culture of continuous quality improvement at the facilities [32]. A similar initiative for public health facilities in India has been shown to improve quality of care as well as improve maternal and neonatal outcomes when implemented at a large scale [52]. Thus, in tandem with ongoing national initiatives to improve service delivery and quality of care in the public sector [5–11], initiatives for the private sector such as Manyata could support the attainment of universal health coverage.

### Strengths and limitations

To the best of our knowledge, this is the first descriptive study of a public health initiative for private-sector healthcare facilities offered by a medical professional association to improve quality of care and achieve accreditation. The study is also notable as it describes performance gaps in small-scale private healthcare facilities, most of which had fewer than 50 beds.

However, as a retrospective analysis of programmatic data, the study has certain limitations. As the facilities included in the analysis undertook NABH assessments on their own volition and were not selected randomly, they may not be representative of the private health sector in India. The sample size is small, so the findings may not be generalizable to private facilities across the country. The assessment findings should also be interpreted with caution, considering the unique assessment methodology of NABH. The analysis of non-compliances based on programmatic experience lacks the methodological rigor that is required in classical research.

## Conclusion

This paper yields valuable insights related to accreditation experiences of private healthcare facilities in the Indian setting. It demonstrates the feasibility of implementing a quality improvement program for small-scale private healthcare facilities in order to help them achieve accreditation. These findings are also of great value for the owners and managers of private healthcare facilities who are interested in applying for accreditation. They should focus on ensuring proper documentation of hospital policies and case records, and on orienting staff to perform the necessary documentation according to standards of care. It also points towards potential shortcomings in the NABH assessment methodology, which may require further introspection. Accreditation results are only as good as the tools and methods used to generate them. The findings are of broader relevance to policymakers, program managers, governments, donors, and implementing organizations that are designing programs targeting the private healthcare sector in India as well as similar settings globally.

## Electronic supplementary material

Below is the link to the electronic supplementary material.

Supplementary Material 1

Supplementary Material 2

Supplementary Material 3: Table 1. List of objective elements which were achieved by all the facilities. Table 2. List of objective elements which were most commonly not achieved by the facilities (ie. the most common non-compliances). Table 3. Distribution of non-compliances by facility characteristics

## Data Availability

De-identified dataset containing the facilities’ baseline and NABH assessment results will be made available upon reasonable request to the corresponding author.

## List of abbreviations

FOGSI: Federation of Obstetric and Gynaecological Societies of India
IRB: Institutional Review Board
ISQua: International Society for Quality in Healthcare
NABH: National Accreditation Board for Hospitals and Healthcare Providers
SHCOs: Small health care organizations
SOPs: Standard operating procedures

## References

[1] Mate KS, Rooney AL, Supachutikul A, Gyani G (2014). Accreditation as a path to achieving universal quality health coverage. Global Health.

[2] Araujo CAS, Siqueira MM, Malik AM (2020). Hospital accreditation impact on healthcare quality dimensions: a systematic review. Int J Qual Health Care.

[3] International Society for Quality in Healthcare (ISQua). Available from: https://isqua.org/ [accessed 25 September 2022].

[4] Government of India. Ministry of Health and Family Welfare. National Health Policy 2017. Available from: https://www.nhp.gov.in/nhpfiles/national_health_policy_2017.pdf [accessed 25 September [accessed 25 September 2022].022]

[5] Government of India. Ministry of Health and Family Welfare. Implementation Guidebook for Kayakalp. Available from: http://www.nhm.gov.in/images/pdf/in-focus/Implementation_Guidebook_for_Kayakalp.pdf [accessed 25 September 2022].

[6] Government of India. Ministry of Health and Family Welfare. Maternal Health Division. DAKSHATA: Empowering Providers for Improved MNH Care during Institutional Deliveries: A strategic initiative to strengthen quality of intra- and immediate postpartum care. Operational Guidelines. April 2015. Available from: https://nhm.gov.in/WriteReadData/l892s/81164783601523441220.pdf [accessed 25 September 2022].

[7] Government of India. National Health Systems Resource Centre. National Quality Assurance Standards. Available from: https://qps.nhsrcindia.org/national-quality-assurance-standards [accessed 25 September 2022].

[8] Government of India. Ministry of Health and Family Welfare. Labour Room Quality Improvement Initiative. 2017. Available from: https://nhm.gov.in/New_Updates_2018/NHM_Components/RMNCH_MH_Guidelines/LaQshya-Guidelines.pdf [accessed 25 September 2022].

[9] Lahariya C, Sharma S, Agnani M, de Graeve H, Srivastava JN, Bekedam H (2022). Attributes of Standard Treatment Guidelines in clinical settings and Public Health Facilities in India. Indian J Community Med.

[10] Lahariya C (2020). Health & Wellness Centers to strengthen primary Health Care in India: Concept, Progress and Ways Forward. Indian J Pediatr.

[11] Lahariya C (2018). Ayushman Bharat’ program and Universal Health Coverage in India. Indian Pediatr.

[12] Rao M, Rao KD, Kumar AKS, Chatterjee M, Sundararaman T (2011). Human resources for health in India. Lancet.

[13] Government of India. Ministry of Statistics and Program Implementation. National Sample Survey Office. Health in India. NSS 71st Round (January – June 2014). Available from: http://mospi.nic.in/sites/default/files/publication_reports/nss_rep574.pdf [accessed 25 September 2022].

[14] Sharma G, Powell-Jackson T, Haldar K, Bradley J, Filippi V (2017). Quality of routine essential care during childbirth: clinical observations of uncomplicated births in Uttar Pradesh, India. Bull World Health Organ.

[15] Tripathi S, Srivastava A, Memon P, Nair TS, Bhamare P, Singh D (2019). Quality of maternity care provided by private sector healthcare facilities in three states of India: a situational analysis. BMC Health Serv Res.

[16] Iyer V, Mavalankar D, Tolhurst R, De Costa A (2020). Perceptions of quality of care during birth at private Chiranjeevi facilities in Gujarat: lessons for Universal Health Coverage. Sex Reprod Health Matters.

[17] Bhate-Deosthali P, Khatri R, Wagle S (2011). Poor standards of care in small, private hospitals in Maharashtra, India: implications for public–private partnerships for maternity care. Reprod Health Matters.

[18] Karvande S, Sonawane D, Chavan S, Mistry N (2016). What does quality of care mean for maternal health providers from two vulnerable states of India? Case study of Bihar and Jharkhand. J Health Popul Nutr.

[19] Vora KS, Mavalankar DV. Quality of reproductive and child health care in the private sector in India: issues and options. Int J Sci Res. 2014;3(8):181–186. Available from: https://www.ijsr.net/archive/v3i8/MDIwMTUyMTY=.pdf [accessed 10 April 2023].

[20] Sharma G, Penn-Kekana L, Halder K, Filippi V (2019). An investigation into mistreatment of women during labour and childbirth in maternity care facilities in Uttar Pradesh, India: a mixed methods study. Reprod Health.

[21] Verma A, Cleland J (2022). Is newborn survival influenced by place of delivery? A comparison of home, public sector and private sector deliveries in India. J Biosoc Sci.

[22] Srivastav N, Pant L, Priya A, Coffey D. Understanding High Mortality among Private Facility Births in Rural Uttar Pradesh. Econ Polit Wkly. 2023;58(10). Available from: https://www.epw.in/journal/2023/10/special-articles/understanding-high-mortality-among-private.html [accessed 10 April 2023].

[23] World Health Organization, Organisation for Economic Co-operation and Development, and The World Bank. Delivering quality health services: a global imperative for universal health coverage. Available from: https://apps.who.int/iris/handle/10665/272465 [accessed 10 April 2023].

[24] World Health Organization. Health care accreditation and quality of care: exploring the role of accreditation and external evaluation of health care facilities and organizations. Available from: https://apps.who.int/iris/handle/10665/363528 [accessed 10 April 2023].

[25] Gyani GJ, Krishnamurthy B. The National Accreditation Board for Hospital and Health Care Providers accreditation programme in India. World Hosp Health Serv. 2014;50(1):9–12. Available from: https://www.researchgate.net/publication/263293004_The_National_Accreditation_Board_for_Hospital_and_Health_Care_Providers_accreditation_programme_in_India [accessed 25 September 2022]. 24938026

[26] David SNJ, Valas S (2017). National Accreditation Board for Hospitals and Healthcare Providers (NABH) Standards: a review. Curr Med Issues.

[27] National Accreditation Board for Hospitals & Healthcare Providers (NABH) [Internet]. Available from: https://www.nabh.co/SHCO-EntryLevel.aspx [accessed 25 September 2022].

[28] National Accreditation Board for Hospitals & Healthcare Providers (NABH) [Internet]. Available from: https://www.nabh.co/Hospital-EntryLevel.aspx [accessed 25 September 2022].

[29] Insurance Regulatory and Development Authority of India. Notification [Internet]. Available from: https://nabh.co/Announcement/IRDA.pdf [accessed 25 September 2022].

[30] Government of India. Ministry of Health and Family Welfare. Ayushman Bharat Pradhan Mantri Jan Arogya Yojana (AB-PMJAY) [Internet]. Available from: https://www.pib.gov.in/PressReleasePage.aspx?PRID=1738169 [accessed 10 April 2023].

[31] Yadav V, Kumar S, Balasubramaniam S, Srivastava A, Pallipamula S, Memon P (2017). Facilitators and barriers to participation of private sector health facilities in government-led schemes for maternity services in India: a qualitative study. BMJ Open.

[32] Delaney MM, Usmanova G, Nair TS, Neergheen VL, Miller K, Fishman E (2022). Does Quality Certification work? An Assessment of Manyata, a Childbirth Quality Program in India’s private Sector. Glob Health Sci Pract.

[33] National Accreditation Board for Hospitals & Healthcare Providers (NABH). [Internet]. Available from: https://www.nabh.co/EntryLevelSHCO_AssessorGuide.aspx [accessed 25 September 2022].

[34] Ogrinc G, Davies L, Goodman D, Batalden P, Davidoff F, Stevens D (2016). SQUIRE 2.0 (Standards for QUality Improvement Reporting Excellence): revised publication guidelines from a detailed consensus process: Table 1. BMJ Qual Saf.

[35] Greenfield D, Braithwaite J (2008). Health sector accreditation research: a systematic review. Int J Qual Health Care.

[36] Woodhead A (2013). Scoping medical tourism and international hospital accreditation growth. Int J Health Care Qual Assur.

[37] Devkaran S, O’Farrell PN, Ellahham S, Arcangel R (2019). Impact of repeated hospital accreditation surveys on quality and reliability, an 8-year interrupted time series analysis. BMJ Open.

[38] Sudha P. Gap Analysis of Major Operation Theatre Complex of a Tertiary Cancer Centre against NABH Accreditation Standards. Kerala Med J. 2015;8(3):84–88. Available from: https://www.keralamedicaljournal.com/index.php/KMJ/article/view/372 [accessed 25 September 2022].

[39] Jagadale SA, Kapurkar KS, Babar R. A study to evaluate change in attitude of medical staff towards acceptance of NABH in Krishna Hospital, Karad. J Evol Med Dent Sci. 2016;5(84):6244–6248. Available from: https://www.jemds.com/data_pdf/1_Kavitha--.pdf [accessed 25 September 2022].

[40] El-Jardali F, Hemadeh R, Jaafar M, Sagherian L, El-Skaff R, Mdeihly R et al. The impact of accreditation of primary healthcare centers: successes, challenges and policy implications as perceived by healthcare providers and directors in Lebanon. BMC Health Serv Res 2014 Feb 25;14:86. doi: 10.1186/1472-6963-14-86. 10.1186/1472-6963-14-86 PMC3946059 24568632

[41] Ajay K, Poka A, Narayan M (2021). Impact of accreditation on documentation and staff perception in the ophthalmology department of an indian medical college. Indian J Ophthalmol.

[42] Ng GKB, Leung GKK, Johnston JM, Cowling BJ (2013). Factors affecting implementation of accreditation programmes and the impact of the accreditation process on quality improvement in hospitals: a SWOT analysis. Hong Kong Med J.

[43] Alkhenizan A, Shaw C (2012). The attitude of health care professionals towards accreditation: a systematic review of the literature. J Family Community Med.

[44] Nandraj S, Khot A, Menon S, Brugha R (2001). A stakeholder approach towards hospital accreditation in India. Health Policy Plan.

[45] Bukonda N, Tavrow P, Abdallah H, Hoffner K, Tembo J (2002). Implementing a national hospital accreditation program: the zambian experience. Int J Qual Health Care.

[46] Yousefinezhadi T, Mosadeghrad AM, Arab M, Ramezani M, Sari AA. An Analysis of Hospital Accreditation Policy in Iran. Iran J Public Health. 2017;46(10):1347–1358. Available from: https://www.ncbi.nlm.nih.gov/pmc/articles/PMC5750346/ [accessed 25 September 2022]. PMC5750346 29308378

[47] Dastur FD. Hospital accreditation: a certificate of proficiency for healthcare institutions. J Assoc Physicians India. 2012;60:12–13. Available from: https://japi.org/article/files/hospital_accreditation_a_certificate_of_proficiency_for_healthcare_institutions.pdf [accessed 25 September 2022]. 23029735

[48] Institute of Medicine (US) Committee on Quality of Health Care in America. To Err is Human: Building a Safer Health System [Internet]. Kohn LT, Corrigan JM, Donaldson MS, editors. Washington (DC): National Academies Press (US). ; 2000. Available from: http://www.ncbi.nlm.nih.gov/books/NBK225182/ [accessed 25 September [accessed 25 September 2022].022] 25077248

[49] O’Leary DS (2000). Accreditation’s role in reducing medical errors. West J Med.

[50] Benova L, Macleod D, Footman K, Cavallaro F, Lynch CA, Campbell OM (2015). Role of the private sector in childbirth care: cross-sectional survey evidence from 57 low- and middle-income countries using demographic and health surveys. Trop Med Int Health.

[51] International Institute for Population Sciences. National Family Health Survey-5 2019–2021 India Fact Sheet. Available from: http://rchiips.org/nfhs/NFHS-5_FCTS/India.pdf [accessed 10 April 2023].

[52] Jain Y, Chaudhary T, Joshi CS, Chotiya M, Sinha B, Nair TS (2022). Improving quality of intrapartum and immediate postpartum care in public facilities: experiences and lessons learned from Rajasthan state, India. BMC Pregnancy Childbirth.

