# Supplementary material for: Implementing a quality improvement initiative for private healthcare facilities to achieve accreditation: experience from India
Source: BMC Health Serv Res. 2023 Jul 27;23:802. doi: 10.1186/s12913-023-09619-w (PMC10375635; doi:10.1186/s12913-023-09619-w)
Supplement: Supplementary file 1 — Supplementary Material 1 [file 12913_2023_9619_MOESM1_ESM.pdf]

Self Assessment Toolkit

Organisation is required to provide self assessment report in the format 'Self Assessment Toolkit' given below. All the entries are to be properly filled up. Regarding scoring following criteria would be applicable.

Compliance to the requirement: 10
Partial compliance to the requirement: 5 (if any of the sample is found to be noncomplying out of total samples selected)
Non-compliance to the requirement: 0
Not Applicable: NA

Evaluation Criteria

- Overall score of minimum 50% in all standards
- Overall score of minimum 50% in each chapter

(Name & Address of the SHCO)

SELF ASSESSMENT TOOLKIT

Elements			Documentation (Yes/ No)	Implementation (Yes/ No)	Evidence (cross reference to documents/ manuals etc.)	Scores (0/ 5/ 10)
**Chapter 1: ACCESS, ASSESSMENT AND CONTINUITY OF CARE (AAC)**						
**AAC.1: The organization defines and displays the services that it can provide.**						
	a	The services being provided are clearly defined.				
	b	The defined services are prominently displayed.				
	c	The relevant staffs are oriented to these services.				
**AAC.2: The organization has a documented registration, admission and transfer process.**						
	a.	Process addresses registering and admitting out-patients, in-patients and emergency patients.				
	b.	Process addresses mechanism for transfer or referral of patients who do not match the organizational resources.				
**AAC.3 Patients cared for by the organization undergo an established initial assessment.**						
	a.	The organization defines the content of the assessments for in-patients and emergency patients.				
	b.	The organization determines who can perform the assessments.				
	c.	The initial assessment for in-patients is documented within 24 hours or earlier.				
**AAC.4 Patient care is continuous and all patients cared for by the organization undergo a regular reassessment.**						

	a.	During all phases of care, there is a qualified individual identified as responsible for the patient’s care who coordinates the care in all the settings within the organization.				
	b.	All patients are reassessed at appropriate intervals.				
	c.	Staff involved in direct clinical care document reassessments.				
	d.	Patients are reassessed to determine their response to treatment and to plan further treatment or discharge.				
**AAC.5 Laboratory services are provided as per the scope of the hospital's services and laboratory safety requirements.**						
	a.	Scope of the laboratory services are commensurate to the services provided by the organization.				
	b.	Procedures guide collection, identification, handling, safe transportation, processing and disposal of specimens.				
	c.	Laboratory results are available within a defined time frame and critical results are intimated immediately to the concerned personnel.				
	d.	Laboratory personnel are trained in safe practices and are provided with appropriate safety equipment/ devices.				
**AAC.6 Imaging services are provided as per the scope of the hospital's services and established radiation safety programme.**						
	a.	Imaging services comply with legal and other requirements.				
	b.	Scope of the imaging services are commensurate to the services provided by the organization.				
	c.	Imaging results are available within a defined time frame and critical results are intimated immediately to the concerned personnel.				
	d.	Imaging personnel are trained in safe practices and are provided with appropriate safety equipment/ devices.				
**AAC.7 Organization has a defined discharge process.**						
	a.	Process addresses discharge of all patients including Medico-legal cases and patients leaving against medical advice.				

	b.	A discharge summary is given to all the patients leaving the organization (including patients leaving against medical advice).				
	c.	Discharge summary contains the reasons for admission, significant findings, investigation results, diagnosis, procedure performed (if any), treatment given and the patient's condition at the time of discharge.				
	d.	Discharge summary contains follow up advice, medication and other instructions in an understandable manner.				
	e.	Discharge summary incorporates instructions about when and how to obtain urgent care.				
	f.	In case of death the summary of the case also includes the cause of death.				
**Chapter 2: CARE OF PATIENTS (COP)**						
**COP.1: Care of patients is guided by accepted norms and practice.**						
	a	The care and treatment orders are signed and dated by the concerned doctor.				
	b	Clinical Practice Guidelines are adopted to guide patient care wherever possible.				
**COP.2: Emergency services including ambulance are guided by documented procedures and applicable laws and regulations.**						
	a	Documented procedures address care of patients arriving in the emergency including handling of medico-legal cases.				
	b	Staff should be well versed in the care of emergency patients in consonance with the scope of the services of hospital.				
	c	Admission or discharge to home or transfer to another organization is also documented.				
**COP.3: Documented procedures define rational use of blood and blood products.**						
	a	The transfusion services are governed by the applicable laws and regulations.				

	b	Informed consent is obtained for donation and transfusion of blood and blood products.				
	c	Procedure addresses documenting and reporting of transfusion reactions.				
**COP.4: Documented procedures guide the care of patients as per the scope of services provided by hospital in Intensive care and high dependency units.**						
	a	Care of patient is in consonance with the documented procedures.				
	b	Adequate staff and equipment are available.				
**COP.5: Documented procedures guide the care of obstetrical patients as per the scope of services provided by hospital.**						
	a	The organization defines the scope of obstetric services.				
	b	Obstetric patient's care includes regular ante-natal check-ups, maternal nutrition and post-natal care.				
	c	The organization has the facilities to take care of neonates.				
**COP.6: Documented procedures guide the care of paediatric patients as per the scope of services.**						
	a	The organization defines the scope of its paediatric services.				
	b	Provisions are made for special care of children by competent staff.				
	c	Patient assessment includes detailed nutritional growth and immunization assessment.				
	d	Procedure addresses identification and security measures to prevent child/ neonate abduction and abuse.				
	e	The children's family members are educated about nutrition, immunization and safe parenting.				
**COP.7: Documented procedures guide the administration of anaesthesia.**						

	a	There is a documented policy and procedure for the administration of anaesthesia				
	b	All patients for anaesthesia have a pre-anaesthesia assessment by a qualified/trained individual.				
	c	The pre-anaesthesia assessment results in formulation of an anaesthesia plan which is documented.				
	d	An immediate preoperative re-evaluation is documented.				
	e	Informed consent for administration of anaesthesia is obtained by the anaesthetist.				
	f	Anaesthesia monitoring includes regular and periodic recording of heart rate, cardiac rhythm, respiratory rate, blood pressure, oxygen saturation, airway [illegible]				
	g	Each patient’s post-anaesthesia status is monitored and documented.				
**COP.8: Documented procedure guides the care of patients undergoing surgical procedures.**						
	a	Surgical patients have a preoperative assessment and a provisional diagnosis documented prior to surgery.				
	b	An informed consent is obtained by a surgeon prior to the procedure.				
	c	Documented procedure addresses the prevention of adverse events like wrong site, wrong patient and wrong surgery.				
	d	Qualified persons are permitted to perform the procedures that they are entitled to perform.				
	e	The operating surgeon documents the operative notes and post-operative plan of care.				
	f	The operation theatre is adequately equipped and monitored for infection control practices.				
**Chapter 3: MANAGEMENT OF MEDICATION (MOM)**						
**MOM.1: Documented procedures guide the organization of pharmacy services and usage of medication.**						

	a	Documented procedure shall incorporate purchase, storage, prescription and dispensation of medications.				
	b	These comply with the applicable laws and regulations.				
	c	Sound alike and look alike medications are stored separately.				
	d	Beyond expiry date medications are not stored/used.				
	e	Documented procedures address procurement and usage of implantable prosthesis.				
**MOM.2: Documented procedure guides the prescription of medications.**						
	a	The organization determines who can write orders.				
	b	Orders are written in a uniform location in the medical records.				
	c	Medication orders are clear, legible, dated and signed.				
	d	The organization defines a list of high risk medication & process to prescribe them.				
**MOM.3: Policies and procedures guide the safe dispensing of medications.**						
	a	Medications are checked prior to dispensing including expiry date to ensure that they are fit for use.				
	b	High risk medication orders are verified prior to dispensing.				
**MOM.4: There are defined procedures for medication administration**						
	a	Medications are administered by trained personnel.				
	b	High risk medication orders are verified prior to administration, medication order including patient, dosage, route and timing are verified.				
	c	Prepared medication is labelled prior to preparation of a second drug.				

	d	Medication administration is documented.				
	e	A proper record is kept of the usage, administration and disposal of narcotics and psychotropic medications.				
**MOM.5: Adverse drug events are monitored.**						
	a	Adverse drug events are defined and monitored.				
	b	Adverse drug events are documented and reported within a specified time frame.				
**Chapter 4: PATIENT RIGHTS AND EDUCATION (PRE)**						
**PRE.1: Patient rights are documented displayed and support individual beliefs, values and involve the patient and family in decision making processes.**						
	a	Patient rights include respect for personal dignity and privacy during examination, procedures and treatment.				
	b	Patient rights include protection from physical abuse or neglect.				
	c	Patient rights include treating patient information as confidential.				
	d	Patient rights include obtaining informed consent before carrying out procedures.				
	e	Patient rights include information on how to voice a complaint.				
	f	Patient rights include information on the expected cost of the treatment.				
	g	Patient has a right to have an access to his / her clinical records.				
**PRE.2: Patient and families have a right to information and education about their healthcare needs.**						
	a	Patients and families are educated on plan of care, preventive aspects, possible complications, medications, the expected results and cost as applicable.				

	b	Patients are taught in a language and format that they can understand.				
**Chapter 5: HOSPITAL INFECTION CONTROL (HIC)**						
**HIC.1: The hospital has an infection control manual, which is periodically updated and conducts surveillance activities.**						
	a	It focuses on adherence to standard precautions at all times.				
	b	Cleanliness and general hygiene of facilities will be maintained and monitored.				
	c	Cleaning and disinfection practices are defined and monitored as appropriate.				
	d	Equipment cleaning, disinfection and sterilization practices are included.				
	e	Laundry and linen management processes are also included.				
**HIC.2: The hospital takes actions to prevent or reduce the risks of Hospital Associated Infections (HAI) in patients and employees.**						
	a	Hand hygiene facilities in all patient care areas are accessible to health care providers.				
	b	Adequate gloves, masks, soaps, and disinfectants are available and used correctly.				
	c	Appropriate pre and post exposure prophylaxis is provided to all concerned staff members.				
**HIC.3: Bio-medical Waste (BMW) management practices are followed.**						
	a	The hospital is authorized by prescribed authority for the management and handling of bio-medical waste.				
	b	Proper segregation and collection of bio-medical waste from all patient care areas of the hospital is implemented and monitored.				
	c	Bio-medical waste treatment facility is managed as per statutory provisions (if in-house) or outsourced to authorized contractor(s).				

	d	Requisite fees, documents and reports are submitted to competent authorities on stipulated dates.				
	e	Appropriate personal protective measures are used by all categories of staff handling bio-medical waste.				
**Chapter 6: CONTINUOUS QUALITY IMPROVEMENT (CQI)**						
**CQI.1: There is a structured quality improvement and continuous monitoring programme in the organization.**						
	a	There is a designated individual for coordinating and implementing the quality improvement programme.				
	b	The quality improvement programme is a continuous process and updated at least once in a year.				
	c	Hospital Management makes available adequate resources required for quality improvement programme.				
**CQI.2: The organization identifies key indicators to monitor the structures, processes and outcomes which are used as tools for continual improvement.**						
	a	Organization shall identify the appropriate key performance indicators in both clinical and managerial areas.				
	b	These indicators shall be monitored.				
**Chapter 7: RESPONSIBILITIES OF MANAGEMENT (ROM)**						
**ROM.1: The responsibilities of the management are defined.**						
	a	The organization has a documented organogram.				
	b	The organization is registered with appropriate authorities as applicable.				
	c	The organization has a designated individual(s) to oversee the hospital wide safety programme.				

**ROM.2: The organization is managed by the leaders in an ethical manner.**						
	a	The management makes public the mission statement of the organization.				
	b	The leaders/management guide the organization to function in an ethical manner.				
	c	The organization discloses its ownership.				
	d	The organization's billing process is accurate and ethical.				
**Chapter 8: FACILITY MANAGEMENT AND SAFETY (FMS)**						
**FMS.1: The organization's environment and facilities operate to ensure safety of patients, their families, staff and visitors.**						
	a	Internal and External Signage's shall be displayed in a language understood by the patients/ families and communities.				
	b	Maintenance staff is contactable round the clock for emergency repairs.				
	c	The hospital has a system to identify the potential safety and security risks including hazardous materials.				
	d	Facility inspection rounds to ensure safety are conducted periodically.				
	e	There is a safety education programme for relevant staff.				
**FMS.2: The organization has a program for clinical and support service equipment management.**						
	a	The organization plans for equipment in accordance with its services.				
	b	There is a documented operational and maintenance (preventive and breakdown) plan.				
**FMS.3: The organization has provisions for safe water, electricity, medical gas and vacuum systems.**						

	a	Potable water and electricity are available round the clock				
	b	Alternate sources are provided for in case of failure and tested regularly.				
	c	There is a maintenance plan for medical gas and vacuum systems.				
**FMS.4: The organization has plans for fire and non-fire emergencies within the facilities.**						
	a	The organization has plans and provisions for detection, abatement and containment of fire and non-fire emergencies.				
	b	The organization has a documented safe exit plan in case of fire and non-fire emergencies.				
	c	Staff is trained for their role in case of such emergencies.				
	d	Mock drills are held at least twice in a year.				
**Chapter 9: HUMAN RESOURCE MANAGEMENT (HRM)**						
**HRM.1: There is an on-going programme for professional training and development of the staff.**						
	a	All staff is trained on the relevant risks within the hospital environment.				
	b	Staff members can demonstrate and take actions to report, eliminate/ minimize risks.				
	c	Training also occurs when job responsibilities change/ new equipment is introduced.				
**HRM.2: The organization has a well-documented disciplinary and grievance handling procedure.**						
	a	A documented procedure with regard to these is in place.				
	b	The documented procedure is known to all categories of employees in the organization.				

	c	Actions are taken to redress the grievance.				
**HRM.3: The organization addresses the health needs of the employees.**						
	a	Health problems of the employees are taken care of in accordance with the organization's policy.				
	b	Occupational health hazards are adequately addressed.				
**HRM.4: There is documented personal record for each staff member.**						
	a	Personal files are maintained in respect of all employees.				
	b	The personal files contain personal information regarding the employees qualification, disciplinary actions and health status.				
**Chapter 10: INFORMATION MANAGEMENT SYSTEM (IMS)**						
**IMS.1: The organization has a complete and accurate medical record for every patient.**						
	a	Every medical record has a unique identifier.				
	b	Organisation identifies those authorized to make entries in medical record.				
	c	Every medical record entry is dated and timed.				
	d	The author of the entry can be identified.				
	e	The contents of medical record are identified and documented.				
**IMS.2: The medical record reflects continuity of care.**						

	a	The record provides an up-to-date and chronological account of patient care.				
	b	The medical record contains information regarding reasons for admission, diagnosis and plan of care.				
	c	Operative and other procedures performed are incorporated in the medical record.				
	d	The medical record contains a copy of the discharge note duly signed by appropriate and qualified personnel.				
	e	In case of death, the medical records contain a copy of the death certificate indicating the cause, date and time of death.				
	f	Care providers have access to current and past medical record.				
**IMS.3: Documented policies and procedures are in place for maintaining confidentiality, integrity and security of records, data and information.**						
	a	Documented procedures exist for maintaining confidentiality, security and integrity of information.				
	b	Privileged health information is used for the purposes identified or as required by law and not disclosed without the patient's authorisation.				
**IMS.4: Documented procedures exist for retention time of records, data and information.**						
	a	Documented procedures are in place on retaining the patient’s clinical records, data and information.				
	b	The retention process provides expected confidentiality and security.				
	c	The destruction of medical records, data and information is in accordance with the laid down procedure.				
