# Supplementary material for: Implementing a quality improvement initiative for private healthcare facilities to achieve accreditation: experience from India
Source: BMC Health Serv Res. 2023 Jul 27;23:802. doi: 10.1186/s12913-023-09619-w (PMC10375635; doi:10.1186/s12913-023-09619-w)
Supplement: Supplementary file 3 — Supplementary Material 3: Table 1. List of objective elements which were achieved by all the facilities. Table 2. List of objective elements which were most commonly not achieved by the facilities (ie. the most common non-compliances). Table 3. Distribution of non-compliances by facility characteristics [file 12913_2023_9619_MOESM3_ESM.pdf]

SUPPLEMENTARY MATERIAL

Table 1. List of objective elements which were achieved by all the facilities

Sl.No.	Name of Objective Element	Achievement (% of facilities)
COP.4.a	Care of patient is in consonance with the documented procedures.	100%
COP.8.d	Qualified persons are permitted to perform the procedures that they are entitled to perform.	100%
MOM.1.e	Documented procedures address procurement and usage of implantable prosthesis.	100%
PRE.1.c	Patient rights include treating patient information as confidential.	100%
PRE.1.f	Patient rights include information on the expected cost of the treatment.	100%
PRE.1.g	Patient has a right to have an access to his / her clinical records.	100%
ROM.2.b	The leaders/management guide the organization to function in an ethical manner.	100%
ROM.2.d	The organization's billing process is accurate and ethical.	100%
IMS.1.b	Organization identifies those authorized to make entries in medical record.	100%
IMS.2.c	Operative and other procedures performed are incorporated in the medical record.	100%
IMS.2.f	Care providers have access to current and past medical record.	100%
IMS.3.b	Privileged health information is used for the purposes identified or as required by law and not disclosed without the patient's authorization.	100%

Table 2. List of objective elements which were most commonly not achieved by thefacilities (ie. the most common non-compliances)

Sl.No.	Name of Objective Element	Achievement (% of facilities)
MOM.2.c	Medication orders are clear, legible, dated and signed.	36%
IMS.1.d	The author of the entry can be identified.	39%
AAC.7.e	Discharge summary incorporates instructions about when and how to obtain urgent care.	39%
COP.7.e	Informed consent for administration of anaesthesia is obtained by the anaesthetist.	39%
AAC.3.a	The organization defines the content of the assessments for in-patients and emergency patients.	43%
AAC.5.c	Laboratory results are available within a defined time frame and critical results are intimated immediately to the concerned personnel.	46%
COP.8.b	An informed consent is obtained by a surgeon prior to the procedure.	46%
IMS.1.c	Every medical record entry is dated and timed.	50%
CQI.2.a	Organization shall identify the appropriate key performance indicators in both clinical and managerial areas.	50%
COP.7.d	An immediate preoperative re-evaluation is documented.	50%

Table 3. Distribution of non-compliances by facility characteristics

Facility characteristics		Number of non-compliances (% of Total)										Total number of non-compliances	Average non-compliances per facility
		Chapter 1	Chapter 2	Chapter 3	Chapter 4	Chapter 5	Chapter 6	Chapter 7	Chapter 8	Chapter 9	Chapter 10		
State	Jharkhand	28 (25%)	26 (24%)	14 (13%)	3 (3%)	7 (6%)	6 (5%)	3 (3%)	10 (9%)	4 (4%)	9 (8%)	110	22
	Maharashtra	34 (20%)	31 (18%)	27 (16%)	2 (1%)	14 (8%)	5 (3%)	4 (2%)	24 (14%)	9 (5%)	22 (13%)	172	22
	Uttar Pradesh	94 (17%)	139 (25%)	78 (14%)	13 (2%)	35 (6%)	22 (4%)	21 (4%)	59 (11%)	46 (8%)	42 (8%)	549	37
Number of beds	≤50	141 (19%)	168 (22%)	108 (14%)	16 (2%)	51 (7%)	31 (4%)	25 (3%)	83 (11%)	58 (8%)	67 (9%)	748	30
	>50	15 (18%)	28 (34%)	11 (13%)	2 (2%)	5 (6%)	2 (2%)	3 (4%)	10 (12%)	1 (1%)	6 (7%)	83	28
Monthly delivery load	<20	40 (17%)	59 (25%)	35 (15%)	2 (1%)	18 (8%)	8 (3%)	9 (4%)	23 (10%)	22 (9%)	21 (9%)	237	26
	20-50	82 (19%)	95 (22%)	62 (14%)	14 (3%)	23 (5%)	22 (5%)	15 (3%)	50 (11%)	35 (8%)	40 (9%)	438	31
	>50	34 (22%)	42 (27%)	22 (14%)	2 (1%)	15 (10%)	3 (2%)	4 (3%)	20 (13%)	2 (1%)	12 (8%)	156	31
Type of facility	Exclusive maternity facility	66 (19%)	65 (18%)	57 (16%)	8 (2%)	27 (8%)	15 (4%)	9 (3%)	41 (12%)	26 (7%)	38 (11%)	352	32
	Multi-specialty facility	90 (19%)	131 (37%)	62 (13%)	10 (2%)	29 (6%)	18 (4%)	19 (4%)	52 (11%)	33 (7%)	35 (7%)	479	28
